# A Novel Method to Detect 3D Mandibular Changes Related to Soft-Diet Feeding

**DOI:** 10.3389/fphys.2017.00567

**Published:** 2017-08-14

**Authors:** Kana Kono, Chihiro Tanikawa, Takeshi Yanagita, Hiroshi Kamioka, Takashi Yamashiro

**Affiliations:** ^1^Department of Orthodontics, Okayama University Hospital Okayama, Japan; ^2^Department of Orthodontics and Dentofacial Orthopedics, Graduate School of Dentistry, Osaka University Osaka, Japan; ^3^Graduate School of Medicine, Dentistry and Pharmaceutical Sciences, Okayama University Okayama, Japan

**Keywords:** 3D, CT, soft food, mice, mandible, morphological change

## Abstract

Craniofacial morphology varies among individuals, which is regulated by the interaction between genes and the environment. Soft-diet feeding is a widely-used experimental model for studying the association between the skeletal morphology and muscle-related loading on the bone. Traditionally, these studies have been based on linear and angular measurements provided on two-dimensional (2D) radiographs in the lateral view. However, 2D observation is based on simplification of the anatomical structures and cannot detect three-dimensional (3D) changes in detail. In this study, we newly developed a modified surface-based analysis with micro-3D computed tomography (CT) to examine and detect the 3D changes in the mandible associated with soft-diet feeding. Mice at 3 weeks of age were fed a powdered soft-diet (SD) or hard-diet (HD) of regular rodent pellets until 9 weeks of age. Micro-CT images were taken at age 9 weeks to reconstruct the anatomical architecture images. A computer-generated averaged mandible was superimposed to directly visualize the morphological phenotypes. Gross observation revealed the apparent changes at the posterior body of the mandible, the angular process and the condyle between HD and SD mice. Significant differences in the mapping indicated the regions of significant displacement in the SD mice over the averaged 3D image of the HD mice. This map revealed that vertical displacement was most evident in 3D changes. We also noted a combination of vertical, transverse and anteroposterior directions of displacement in the condylar growth, resulting in complicated shape changes in the whole condylar process in SD mice. In contrast, transverse displacement was more significant in the coronoid process. The map analysis further showed the significant outward displacement of the inner surface of the alveolar process, which consequently resulted in thinning of the alveolar process.

## Introduction

Craniofacial morphology is the result of a complex network of interactions between the genotype and the environment and the mandibular structure adapts according to the physiological loading during mastication (Klingenberg and Leamy, 2001). Malocclusion is more prevalent in modern populations than in ancient ones. The modern diet, characterized by softer and/or processed foods, may contribute to reduced chewing activity and a decreased masticatory function have been linked with an increased prevalence of malocclusion and craniofacial deficiency in humans (Corruccini, 1984; Larsson et al., 2005).

Previous studies have shown that a soft-food diet resulted in a posteriorly rotated mandible, reduced ramus size, more posteriorly directed growth of the condyle, and a shorter vertically angular process (Bouvier and Hylander, 1984; Kiliaridis et al., 1985; Yamada and Kimmel, 1991; Maki et al., 2002; Anderson et al., 2014). Soft diet leads to reduced masticatory muscle strength and decreased loading applied to the mandible, which can affect the bone modeling specifically in the regions where the masticatory muscle attaches (Mavropoulos et al., 2004; Hichijo et al., 2014, 2015). These findings provided solid evidence that the masticatory muscle function influences the mandibular growth pattern and thus the morphology of the mandible. However, these studies were based on linear and angular measurements obtained on two-dimensional (2D) radiographs. The rodent and human craniofacial structure is complicated in shape, and 2D observations insufficiently display the anatomical structures by representing them as sets of landmarks.

In addition, a 2D radiograph analysis in smaller animals, e.g., young mice, is difficult due to unstable head positioning and mandible orientation in comparison to rats or larger animals. Indeed, in the aforementioned 2D studies identifying the bone phenotypes associated with soft-diet feeding, they used mice 6 months old, or rats only 8 weeks old. Young mice have advantages over other mammals, as genetically modified mice are commonly used as animal models of human diseases.

These limitations are currently overcome by acquiring three-dimensional (3D) micro-computed tomography (CT) images, which permits capturing the shapes of the subjects with high resolution and provide methods to visualize, quantify and compare the surface morphology of the materials in 3D (Nakano et al., 2003). Micro-CT generates reconstructed 3D models in voxels for qualitative 3D morphological observation, and then the bone surface voxel can be extracted and converted to coordinates for quantitative morphometric analysis (Swain and Xue, 2009). However, most previous studies using 3D micro-CT performed 2D observation on a reconstructed slice or measurement among the reference points placed on reconstructed models (Mavropoulos et al., 2004; Swain and Xue, 2009; Enomoto et al., 2010; Saito et al., 2011; Goto et al., 2015). While 3D observation using micro-CT provided more accurate data than previous 2D studies had, a conventional 3D analysis did not show any additional advantages for evaluating any possible deviations in the mandibular shape.

3D volumetric images are first converted into 3D surface models, and then the Standard Tessellation Language software program (STL; a triangulated mesh representation of 3D images) makes it possible to parameterize the entire curved surface (Gelaude et al., 2008). In our previous studies, wire mesh fitting based on the anatomical landmarks were applied to the STL data, which allowed for the detection of deviations in the parameters describing the facial morphological phenotypes among males and females (Tanikawa et al., 2016). In the present study, we first applied wire mesh fitting for a 3D analysis of micro-CT data to evaluate how food consistency influences the whole mandibular morphology. A wire mesh fitting analysis using approximately 10,000 points of wire mesh on the mandibular surface was applied to assess the quantitative topographic variation in the bony surface of the mandible. Our approach clearly visualized the 3D distribution of significant changes on the mandibular surface in response to changes in food consistency.

## Materials and methods

### Animals

Sixteen ICR mice (body weight 7 g, all obtained from Crea Japan, Tokyo, Japan), weaned at postnatal 3 weeks, were randomly divided into groups. The control group was fed a hard diet (HD) of regular rodent pellets (CE-2, Crea Japan) with tap water, and the soft-diet (SD) group was given a powdered diet of the same nutritional value (powdered CE-2) until postnatal 9 weeks. All aspects of animal care and experiments were approved by the Okayama University Committee for Animal Care and Use. All animals, housed three or four to a cage, were fed and watered *ad libitum* and maintained at 23 ± 1°C with a 12-h day/night cycle.

The animal experiments were performed under the research protocol approved by the Animal Research Committee at Okayama University (OKU-2014520). To minimize animal suffering, the number of animals used was based on the minimum required to obtain statistically valid results.

### Micro-CT

Nine mice with SD and 7 with HD were sacrificed and decapitated at postnatal 9 weeks. The heads were fixed in 4% paraformaldehyde (PFA) for 12 h. Micro-CT images were taken with a Ratheta LCT-200 In Vivo Micro-CT Scanner for Small Lab Animals (HITACHI-Aloka, Tokyo, Japan) at 90 kV and 110 mA with a 96 μm slice width, and 1-voxel size at 96 × 96 × 96 μm.

### Segmentation

Surface generation from micro-CT data was performed with 3D Slicer (version 4.5.0-1, http://www.slicer.org) (Kikinis and Pieper, 2011), which is an open-source software platform for the analysis and visualization of CT data. Following data preparation, such as painting, erosion, and dilation, to obtain a mandibular 3D model, semiautomatic segmentation was performed on this platform using a region-growing algorithm called GrowCut (Supplementary Figure 1).

### Identification of the landmarks

The positions of 69 landmarks [36 landmarks were defined in previous studies (Kiliaridis et al., 1985; Odman et al., 2008; Boell and Tautz, 2011) and 33 landmarks were newly defined in the present study (See Supplementary Figure 2)] were identified by a visual inspection of the image and digitized using a computer mouse cursor and HBM-Rugle (Medic Engineering Co, Kyoto, Japan). For the reliability of the identification of the landmarks, please see Supplementary Figure 1. The 3D images were imported into the new coordinate system for standardization based on the aforementioned landmarks (Supplementary Figure 2).

### Homogeneous modeling

For each mandibular model, fitting of high-resolution template meshes (Brett et al., 2003) was performed using HBM-Rugle, based on the landmarks assigned to each 3D image. This method automatically generated a homogeneous model consisting of 12,186 points (i.e., nodes of the fitted mesh) on the wire mesh for each model. This technique permits the extraction of relevant surface anatomy from micro-CT data while removing and/or smoothing out non-relevant data, yielding high-resolution, 3D surface data that provide enough detail to facilitate a quantitative assessment while maintaining small file sizes that are easily manipulatable and portable to a range of visualization technologies. The average distance between the points on the meshes was 32 ± 24 μm, which is less than the slice thickness of 96 μm. Thus, the mesh resolution employed was considered adequate for representing the original mandibular shape. The arithmetic means of the coordinate values of each corresponding point on the wire mesh were computed and used to generate the averaged 3D mandibular images for each subject group.

The surface displacement was quantitatively evaluated in each x-, y-, and z-axis in two different ways. The actual displacement and significance of differences were calculated for the 12,186 points on each mesh between the HD and SD groups. The calculated values in millimeters were visualized with color-coding on the computed 3D models of HD mice. Thereafter, the arithmetic means of the coordinate values of each corresponding point on the wire mesh were statistically analyzed for significant differences between the HD and SD groups using a two-sample *t*-test. A significance probability map (Duffy et al., 1981) of the *x*-, *y*-, and *z*-values was generated to visualize these significant differences.

### Accentuated images on SD feeding

To facilitate the quantitative understanding of the 3D phenotypes associated with SD feeding, accentuated averaged mandibles were computed for SD mice to highlight the site-specific impact on these animals (Tanikawa et al., 2016). For details of the calculations, please see Supplementary Figure 2.

## Results

### Average and accentuated average images in SD mice

**Figure 1:**
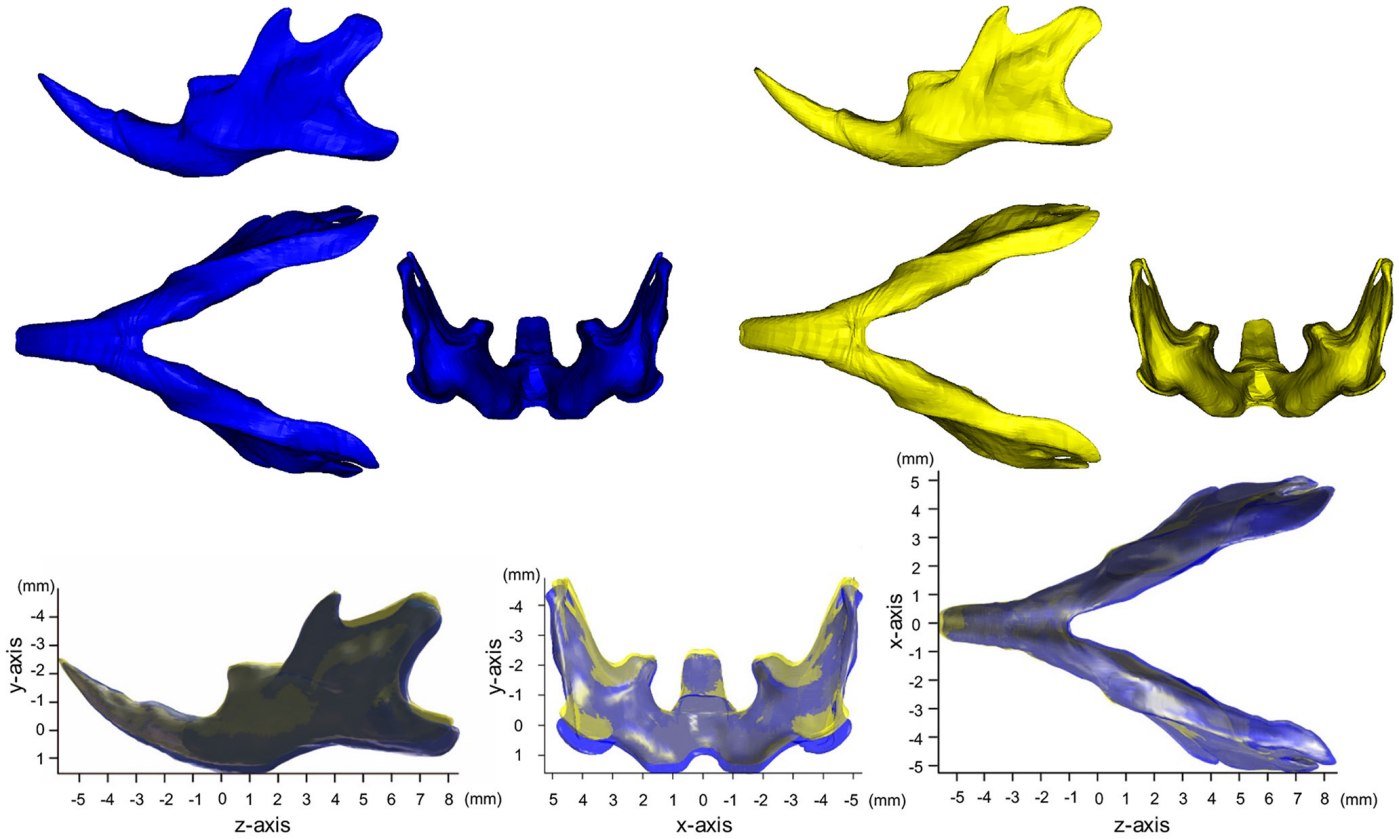
Averaged mandibular surface computed for the SD (Yellow, *n* = 9) and HD groups (Blue, *n* = 7).

**Figure 2:**
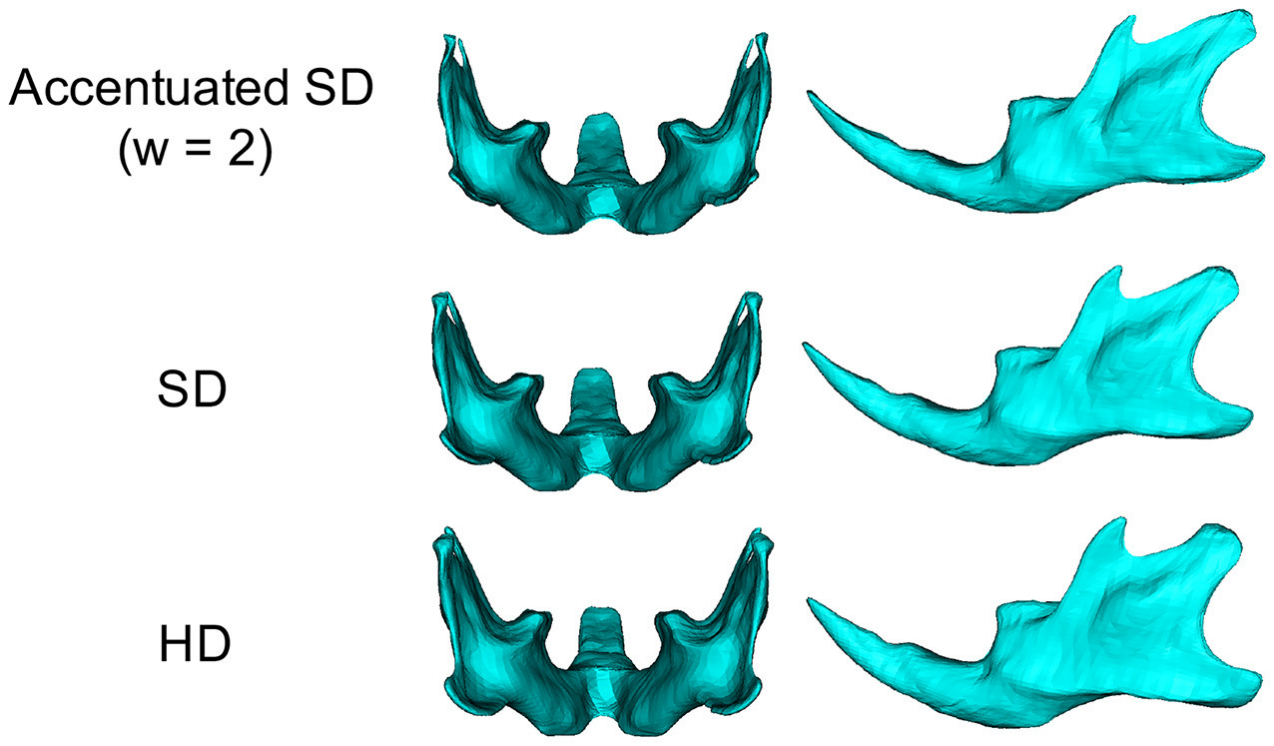
Accentuated SD mandibular surface (**Top**), SD group (**middle**), HD group (**bottom**).

Figure 1 shows the averaged mandibular surface computed for the SD (Yellow in Figure 1) and HD groups (Blue in Figure 1) and superimposed at the mental foramen parallel to the occlusal plane (Kiliaridis et al., 1985; Odman et al., 2008) (For more details, please see Supplementary Figure 3). Figure 2 shows the accentuated images. These images clearly reveal the manner in which SD mandible differs from the HD in 3D. Details of the x-, y-, and z-axis are shown below and in Table 1.

**Table 1:** Result summary of the right halves of the mandible for SD when compared with HD.

	**Surfaces**	**X-axis (Transverse direction)**		**Y-axis (Vertical direction)**		**Z-axis (Antero-posterior direction)**	
		**Displacement**	***p*-value**	**Displacement**	***p*-value**	**Displacement**	***p*-value**
Coronoid process	Lateral (Outer)	Inward	^****^	NS		NS	
	Medial (Inner)		^**^				
	Anterior		^***^				
	Posterior		^***^				
	Superior		^****^				
Condyle	Lateral (Outer)	Inward	^**^	Upward	^**^	NS	
	Medial (Inner)	NS			^*^		
	Anterior	Inward	^*^		^**^		
	Posterior		^*^		^*^		
	Superior		^*^		^*^		
Alveolar process	Lateral (Outer)	NS		Upward	^*^	NS	
	Medial (Inner)	Outward	^****^		^**^		
	Anterior	NS			^**^		
	Posterior	Outward	^****^		^**^		
Inferior part of the mandibular body	Lateral (Outer)	NS		Upward	^****^	NS	
	Medial (Inner)	Inward	^*^		^****^		
	Inferior		^*^		^****^		
Angular process	Lateral (Outer)	Inward	^*^	Upward	^****^	Forward	^**^
	Medial (Inner)	NS			^****^		^*^
	Posterior	Inward	^*^		^****^		^*^
	Superior		^*^		^***^		^**^
	Inferior		^*^		^****^		^*^
Antegonial notch	Lateral (Outer)	NS		Upward	^***^	Forward	^**^
	Medial (Inner)				^**^		^**^
	Posterior				^***^		^***^
Molars	Labial	Inward	^**^	Upward	^***^	NS	
	Lingual		^***^		^**^		
	Occlusal		^**^		^**^		

NS indicates P > 0.05;

* P ≤ 0.05;

** P ≤ 0.01;

*** P ≤ 0.001;

**** *P ≤ 0.0001*.

### Map of significant differences for the X-axis: transverse direction

**Figure 3:**
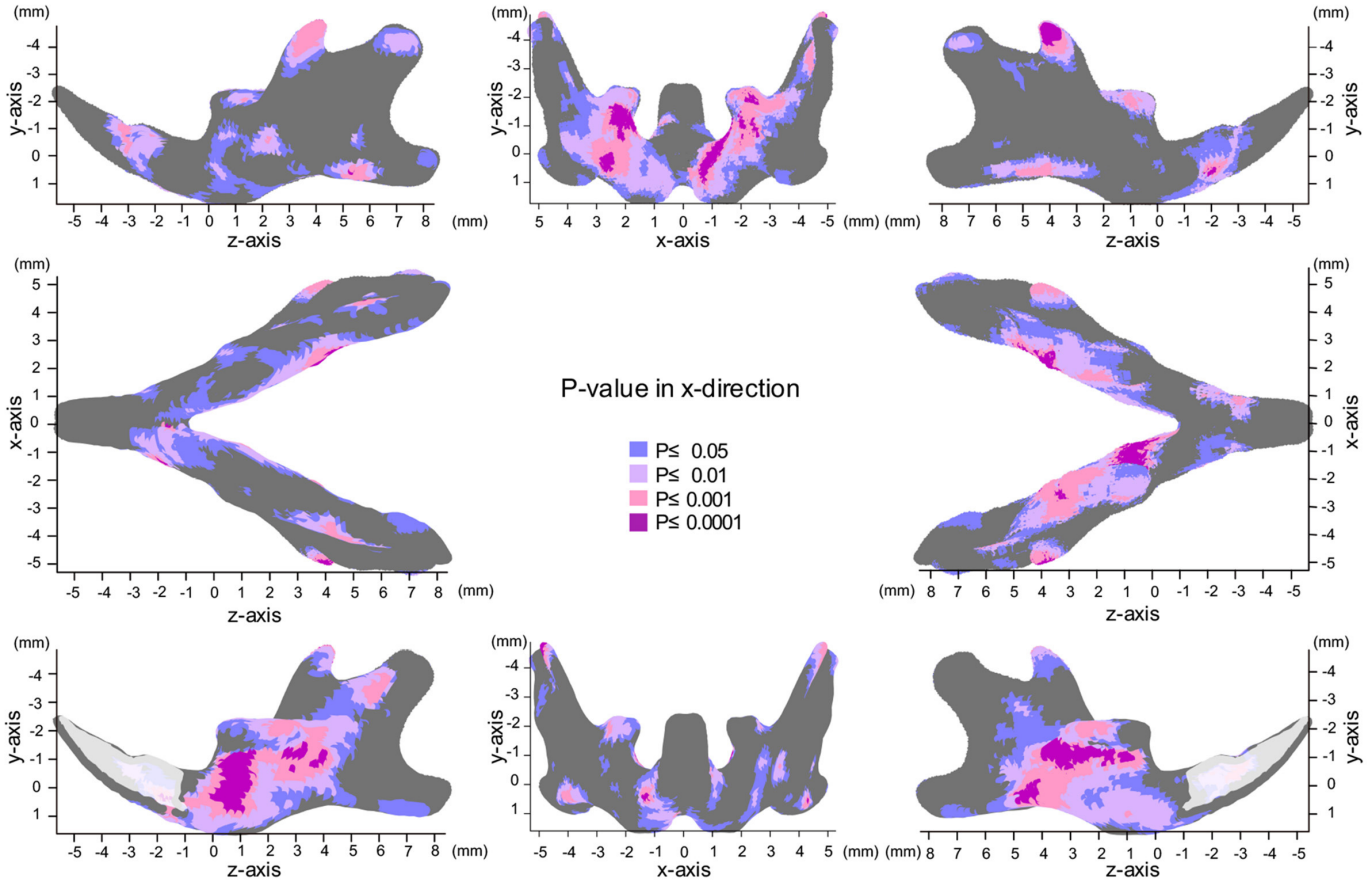
Significance probability map in the x-axis. Blue designates *P* ≤ 0.05; Pale pink, *P* ≤ 0.01; Dark pink, *P* ≤ 0.001; Purple, *P* ≤ 0.0001. Top left indicates left lateral view; top middle, posterior view; top right, right lateral view; middle left, inferior view; middle right, superior view; bottom left, medial view of the right halve of the mandible, and bottom right, medial view of the left halve of the mandible.

**Figure 4:**
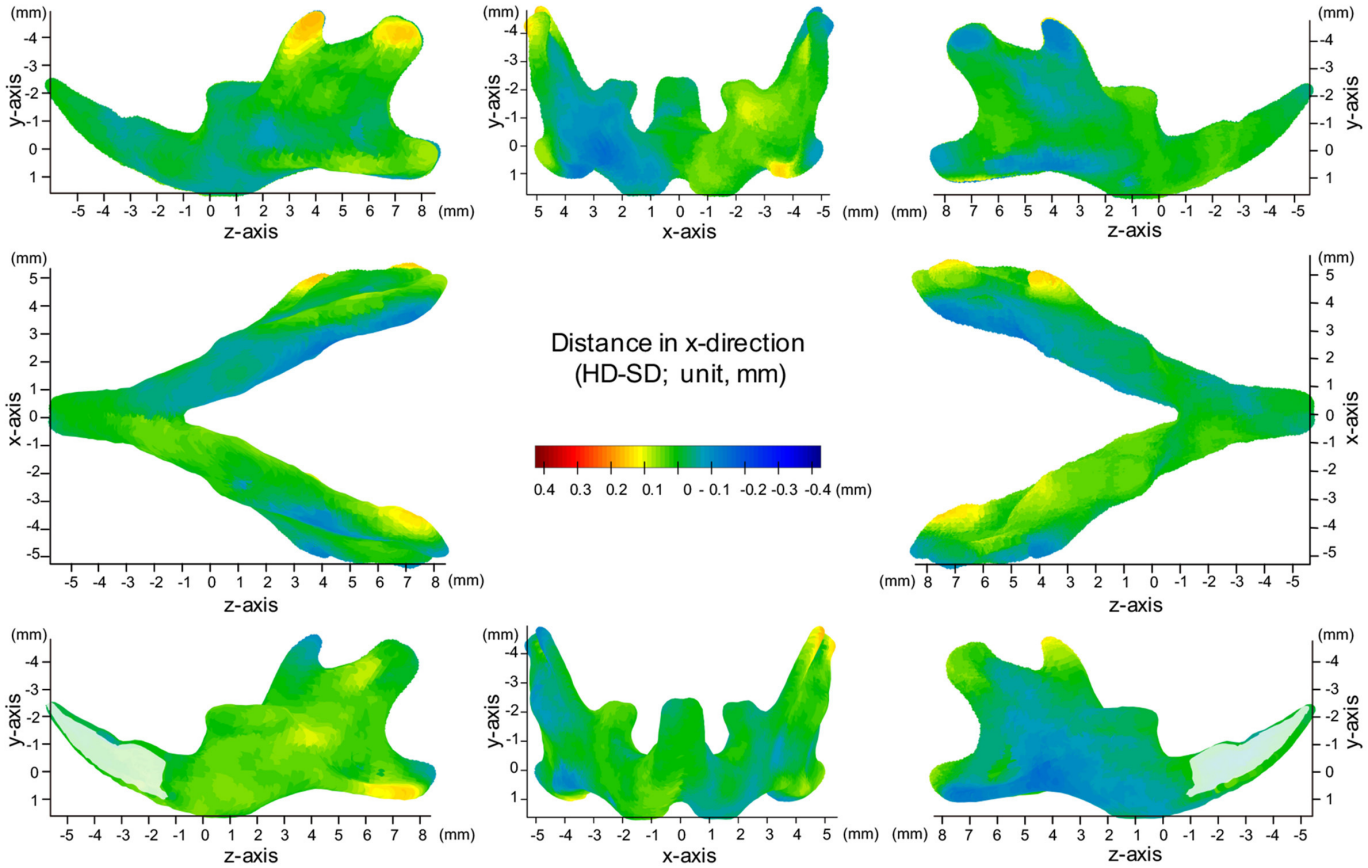
Difference (HD minus SD) in the x-axis. Units: mm. Red indicates that the HD group is greater than the SD group, while blue indicates that the SD group is greater than the HD group.

The most significant changes in the x-axis were observed in the coronoid process (Figures 3, 4, Table 1). The outer surface of the coronoid process was displaced significantly in the inward direction in the SD group, with less-marked displacement also observed in the inner surface. Consequently, the transverse distance between the coronoid process and the width of the coronoid process decreased significantly in comparison to the HD group. Since greater inward displacement of the process was noted at the tip than in other areas, the coronoid process became significantly tipped in the inward direction, as revealed by the significant reduction in the angle of the coronoid process. Like the coronoid process, the outer surface of the condyle was also displaced in the inward direction, whereas the inner surface was not significantly displaced. Consequently, the condyle became significantly thinner in the SD group than in the HD group (Supplementary Figures 4, 5).

In contrast, the inner surface of the alveolar process and of the basal bone supporting the alveolar process was significantly displaced in the outward direction, while the outer surface of these bones was not displaced, leading to a reduction in the transverse width of the alveolar bone and the basal bone at the alveolar bone regions (Supplementary Figure 4C).

### Map of significant differences for the Y-axis: vertical direction

**Figure 5:**
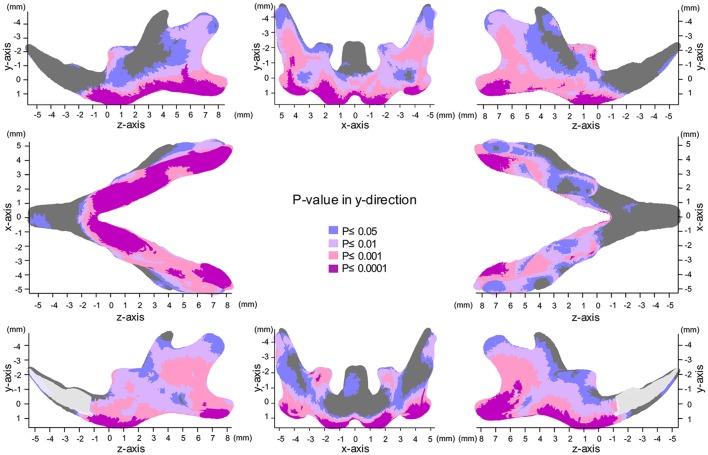
Significance probability map in the y-axis. Blue designates *P* ≤ 0.05; Pale pink, *P* ≤ 0.01; Dark pink, *P* ≤ 0.001; Purple, *P* ≤ 0.0001. Top left indicates left lateral view; top middle, posterior view; top right, right lateral view; middle left, inferior view; middle right, superior view; bottom left, medial view of the right halve of the mandible, and bottom right, medial view of the left halve of the mandible.

**Figure 6:**
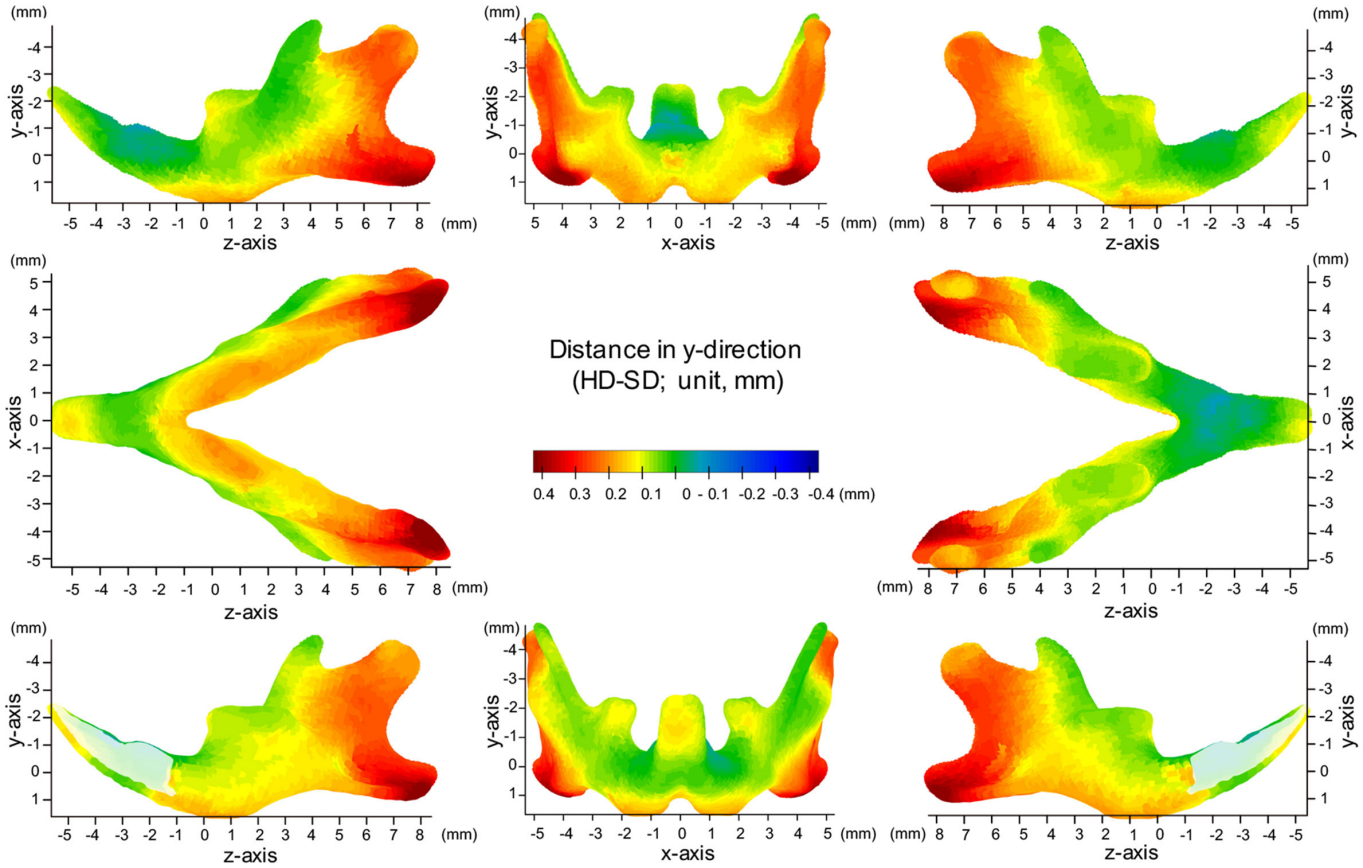
Difference (HD minus SD) in the y-axis. Units: mm. Red indicates that the HD group is greater than the SD group, while blue indicates that the SD group is greater than the HD group.

The significance probability map clearly showed that shape changes associated with SD feeding were most prominently observed in the y-axis among the three dimensions (Figures 5, 6, Table 1). The areas showing significant differences were widely distributed along the inferior surface of the mandible, where remarkable upward displacement was observed due to SD feeding. This led to an apparent decrease in the vertical height of the mandibular corpus (Supplementary Figures 4, 5).

In the angular process, upward displacement was more significant in the inferior surface than in the superior surface, resulting in significant reduction in the vertical width of the angular process (Supplementary Figure 4A). Along the inferior surface of the mandible, upward displacement was most significant at the angular process, causing the mandibular plane angle to steepen. Furthermore, along the inferior border, upward displacement was much less drastic at the antegonial notch, resulting in a relatively flat mandibular surface. In contrast, such upward displacement was not observed in the coronoid process, and less so in the condylar regions. In the condylar process, upward displacement was greater and more significant at the anterior surface than at the posterior surface, resulting in a shallower condyle from the lateral view. These findings can be ascertained more clearly in the accentuated averaged mandibles (Figure 2).

Regarding the vertical positon of the molars, the mean displacement was quite small (less than 0.05 mm), but significant upward displacement, i.e., elongation of the molars, was observed (*P* ≤ 0.05).

### Map of significant differences for the Z-axis: anterior-posterior direction

**Figure 7:**
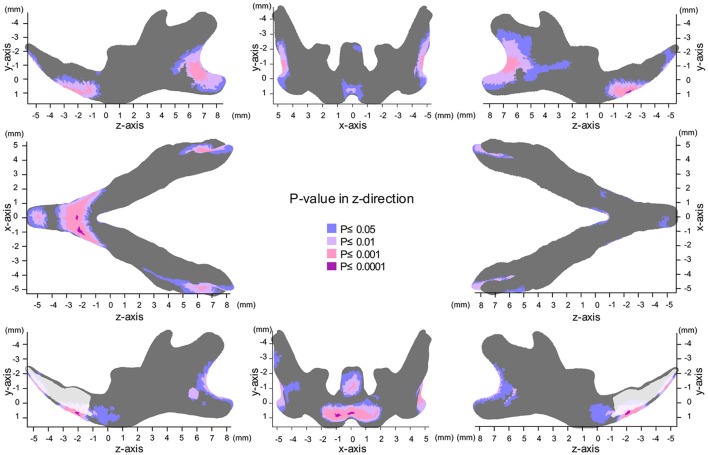
Significance probability map in the z-axis. Blue designates *P* ≤ 0.05; Pale pink, *P* ≤ 0.01; Dark pink, *P* ≤ 0.001; Purple, *P* ≤ 0.0001. Top left indicates left lateral view; top middle, posterior view; top right, right lateral view; middle left, inferior view; middle right, superior view; bottom left, medial view of the right halve of the mandible, and bottom right, medial view of the left halve of the mandible.

**Figure 8:**
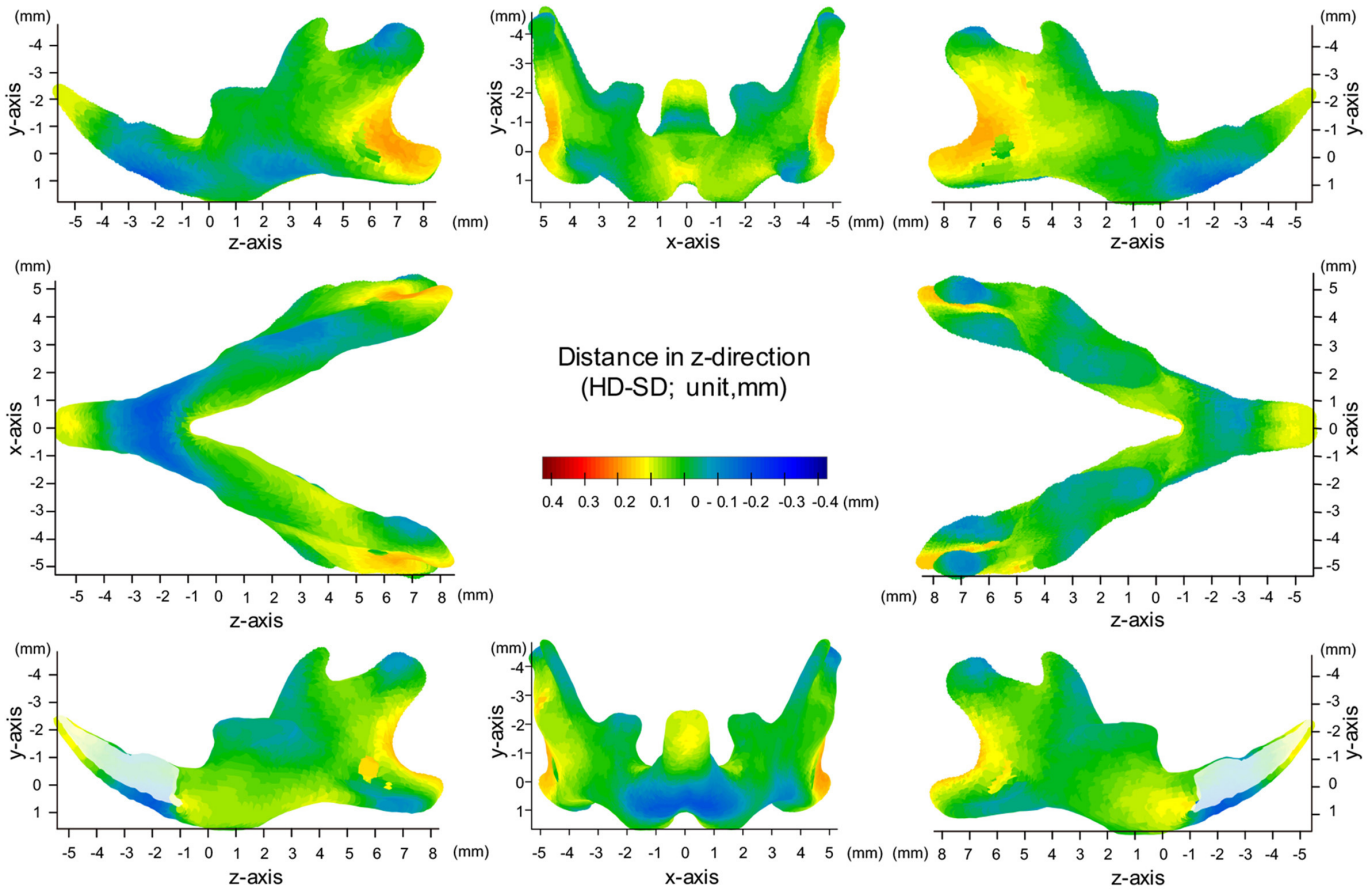
Difference (HD minus SD) in the z-axis. Units: mm. Red indicates that the HD group is greater than the SD group, while blue indicates that the SD group is greater than the HD group.

In the z-axis, significant displacement toward the anterior direction of the angular process and ramus were observed, while the condyle did not show any anterior-posterior displacement (Figures 7, 8, Table 1). These results suggest an anterior-posteriorly smaller angular process and greater gonial angle in the SD group than in the HD group.

## Discussion

In the present study, we applied a surface-based analysis of micro-CT to evaluate the 3D shape changes in the mandible associated with different food consistencies. The present micro-CT data provide highly accurate 3D imaging data, and 12,186 points on the triangulated mesh were fitted to the curved mandibular surface, which enabled the quantitative topographic assessment of the bony surface and reconstruction of the anatomical architecture images with high resolution. In most previous studies, the distance and/or the angle between the corresponding landmarks were measured after the 3D differences in the superimposed models were usually translated into 2D (Mavropoulos et al., 2004; Swain and Xue, 2009; Enomoto et al., 2010; Saito et al., 2011; Goto et al., 2015). In contrast, in our present approach, a computer-generated averaged mandible was superimposed to describe the morphological phenotypes in the mandibular shape, and then variations in the surface morphology were directly visualized and quantified using a 3D image with a map of points of significant difference. In addition, an accentuated averaged SD mandible was developed to confirm and highlight the site-specific skeletal phenotypes associated with SD feeding. This study has shown the novel application of a surface-based analysis for studying the 3D phenotypes of the complicated shapes of the craniofacial structures.

Manipulating raw data using a computer software program carries a risk of possible loss of anatomical detail. A previous study has already validated the procedure for extracting high-resolution surface meshes from micro-CT voxel data (Norman et al., 2014). Another study also verified the absolute 3D accuracy of CT-based bone outer surface meshes using STL (Almukhtar et al., 2014). In our study, micro-CT images were taken with high resolution and a 96 μm slice width. Our experimental conditions in our micro-CT surface-based data analysis using STL format can be used to evaluate the skeletal shape of the rodent mandible.

The craniofacial anatomical changes associated with food consistency have been examined using various experimental animal models, such as monkey (Corruccini and Beecher, 1982), pig (Dias et al., 2011), minipigs (Ciochon et al., 1997) and rats (Kiliaridis et al., 1985, 1999; Kiliaridis, 2006). Mice carry advantages over other mammals because they are less expensive to maintain and genetically modified mice are readily available. However, mice also have disadvantages due to their small size. The smaller size of their experimental samples requires high accuracy in data sampling, especially in the evaluation of topographical changes. In previous 2D analyses using cephalograms, landmarks were difficult to locate compared using CT, and head positioning and mandibular orientation were critical. In addition, 2D cephalometric analyses provided linear and angular measurements, which explains why only a fraction of the changes in the complicated shapes of the mandible were noted. The present combination of micro-CT and an STL analysis overcomes these problems in quantitative morphometric analyses. In the present study, we were able to identify significant shape changes 6 weeks after the start of SD feeding. The duration of the experimental period was also much shorter than in similar previous studies, in which the experimental period lasted about 6 months (Renaud et al., 2010; Anderson et al., 2014). Our novel approach involving a surface-based analysis provided advantages over 2D approaches with respect to the non-destructive and 3D visualization and quantification of the morphology of the experimental materials.

Craniofacial dental and skeletal changes were better detected by superimposing the cephalograms on the stable natural reference structures. The superimposition of the CBCT images of the growing mandible makes it possible to determine the regions of bone apposition and resorption. The reference plane and/or points should be stable, especially when growing experimental animals are used. Previous studies have suggested that the mental foramen and lingual foramen are relatively stable landmarks on the growing mandible because no muscles are attached there (Mavropoulos et al., 2004). However, we found that the bone surface around the lingual foramen was actively modeled and the changes associated with SD in its vertical position were significant. Therefore, only the mental foramen was selected as the point of origin for morphological comparison, and the occlusal plane was additionally used to define the x-z plane.

The patients with hypofunctioning muscles often showed an anterior open bite due to significant posterior tooth eruption, possibly because the reduced mechanical loading on the molars due to SD allows these teeth to further erupt (Lieberman et al., 2004). In our present experiments, the bone surface modeling resulted from a combination of normal growth and the effect of SD. In a previous animal study evaluating the effects of SD on molar eruption, the findings were inconclusive (Maki et al., 2002; Odman et al., 2008). In contrast, our present analysis clearly detected significant elongation of the molars due to SD when evaluated using our reference plane.

The present significance difference map clearly indicated where the SD-induced skeletal shape changes appear on the averaged 3D mandibular images in the x-, y-, and z-axis. The present maps for the x-, y-, and z-axis showed that the y-axis displacement was most evident and the regions showing significant differences in the y-axis were the most widely distributed on the averaged mandibular images, indicating that mandibular growth in the vertical direction was most affected by SD among the 3D changes. The present findings are reasonable because the masticatory muscle runs in the vertical direction, and the direct effect of an SD appears first in the vertical direction.

The present surface map analysis provided for the first time quantitative and comparable 3D data, thereby explaining how the previous findings in the mandibular shape changes were associated with SD. A previous study described SD-induced morphological phenotypes, such as a decreased gonial process, decreased posterior height of the corpus, a steep mandibular plane, a vertically reduced angular process and a shallower antegonial notch (Kiliaridis et al., 1985; Kiliaridis, 2006). Such anatomical changes were clearly explained by region-specific vertical bone modeling on the bone surface, as demonstrated by the significance difference map of y-axis. In other words, our surface-based analysis provided quantitative evidence of how the masticatory activity affects the site-specific response on the bone surface and consequently the 3D complicated growth of the mandible.

With respect to the association between feeding style and craniofacial evolution, our conclusions are consistent with those drawn through mathematical simulations using Finite Element models (Cox et al., 2012; Tsouknidas et al., 2017). For example, it has been shown that rodents have a mechanically efficient morphology of masticatory musculature and skulls to their feeding style, i.e., gnawing or chewing (Cox et al., 2012). Feeding style was also proven to result in varying loading patterns on the mandibles (Tsouknidas et al., 2017), e.g., loads to the masseter ridge, the mental foramen of the mandible, and the temporomandibular joints. As food texture is known to change the feeding style (Peyron et al., 1997), it would be reasonable to state that the food consistency of SD or HD can influence microevolutionary divergence patterns in mandible shape.

The present study additionally suggested that the shape of the condylar process was also modulated by the masticatory muscle in a complicated manner. It has been established that an SD results in a decreased inclination of the condylar process from the lateral view (Kiliaridis, 2006). The present significance map showed that such angular changes in the condylar process were site-specific displacements between the anterior and posterior surface of the condyle.

Furthermore, a previous study demonstrated a reduction in the transverse width of the condyle in SD mice (Kiliaridis et al., 1999). The present significance map showed that such a reduction was caused by the inward displacement of the outer surface of the condyle. Our analysis therefore demonstrated for the first time the inward tipping of the condyle.

As mentioned above, an SD allowed for the elongation of the molars (Odman et al., 2008). The present significance map showed that the outer surface of the alveolar bone or of the basal bone supporting the alveolar process was not affected, while their inner surface was displaced laterally in the outward direction. Thus far, due to the complicated inner surface morphology, it has been quite difficult to accurately measure its distances and/or angles. The findings of our surface-based analysis, however, clearly suggested that the width of the alveolar process and the basal bone significantly decreased with the elongation of the molars.

Our study has several limitations. Firstly, the sample size for our analysis was relatively small; therefore, it is possible that our results would have differed with a larger sample. Secondly, the age and duration of the SD feeding might have an influence on the morphological variety. Therefore we need further research on the impact of the time-related changes of SD feeding. There is also a limitation in our experimental protocol. In original CT images, the internal structure of the craniofacial bone can be visualized, however, only the bony surface information is extracted and evaluated as STL data in our protocol. Hence, the soft diet-induced changes in cortical bone or cartilage thickness, which was demonstrated in the previous study, were excluded from our interest.

## Conclusion

In this study, we presented a novel, accurate superimposition protocol for micro-CT data of the mandibular skeletal structures. Our surface-based analysis detected and visualized the areas showing significant deviation associated with SD on averaged 3D images. The present findings provided 3D quantitative evidence supporting previous findings regarding shape changes with additional novel phenotypes in SD mice. Furthermore, its high accuracy with respect to the quantitative measurement of the bone surface enabled the identification of the anatomical phenotypes in mice in a relatively short period of 6 weeks. In conclusion, our surface-based analysis provided quantitative evidence of how the masticatory activity affects the site-specific response on the bone surface and consequently affects the 3D complicated growth of the mandible in mice.

## Author contributions

KK performed experiments. CT developed analytical methods and analyzed the 3D data. TYan performed experiments. HK gave technical support and conceptual advice. TYam supervised its analysis and edited the manuscript. All authors discussed the results and implications and commented on the manuscript at all stages.

### Conflict of interest statement

The authors declare that the research was conducted in the absence of any commercial or financial relationships that could be construed as a potential conflict of interest.

## Appendix

### Reliability of the identification of landmarks

To determine the intra-observer reliability of the identification of landmarks, the 3D images of 10 subjects were randomly selected, and the coordinate values of the landmarks were determined by the experimenter (KK). The digitization process was then repeated a week later, and the two sets of results were compared. The results showed a mean absolute difference of 45.7 ± 37.4 μm. These values were small compared to the slice thickness (96 μm) of the micro-CT.

### Accentuated images on SD feeding

To quantitatively facilitate our instantaneous and intuitive understanding of the skeletal changes in the mandible associated with SD, accentuated averaged mandibular forms, $\bar{AccA\left(SD_{w}\right)}$, were calculated for the SD and HD groups to highlight the differences between the two subject groups, where

$$\begin{array}{l} \bar{AccA\left(SD_{w}\right)}=\bar{A\left(HD\right)}+w\;\left(\bar{A\left(SD\right)}\;-\bar{A\left(HD\right)}\right)\left(w=2\right) \end{array}$$

and $\bar{A\left(HD\right)}$ and $\bar{A\left(SD\right)}$ are the arithmetic means of the coordinate values for the HD and SD group, respectively, and *w* is the weight value.

### Selection of an appropriate reference plane

The selection of an appropriate reference plane directly affects the interpretation of the 3D skeletal changes associated with different food consistencies. SD results in a decreased occlusal load and masticatory muscle activity, which affects the bone activity directly or indirectly. In a previous 2D study, the occlusal plane was used as reference plane for superimposition in growing experimental animals, and such superimposition clearly demonstrated differences in the shape changes of the mandible between SD and HD rats (Kiliaridis et al., 1985). However, the reference anatomical points should be less influenced by mastication activity during in mandibular growth. Previous studies have used reference planes connecting the mental foramen and lingual foramen (Odman et al., 2008). These landmarks are known to be relatively stable on the growing mandible. In this study, we found that the occlusal plane was significantly displaced upward when evaluated from the mental foramen in SD rats. We also found that the vertical position of the mandibular foramen was unstable in our experimental protocol (Supplementary Figure 3B). Therefore, the averaged 3D images were superimposed at the mental foramen parallel to the occlusal plane (Supplementary Figure 3A)
