# Supplementary material for: A Novel Method to Detect 3D Mandibular Changes Related to Soft-Diet Feeding
Source: Front Physiol. 2017 Aug 14;8:567. doi: 10.3389/fphys.2017.00567 (PMC5557733; doi:10.3389/fphys.2017.00567)
Supplement: Supplementary file 1 [file Image1.PDF]

frontiers

Supplementary Material

A novel method to detect 3D mandibular changes related to soft-diet feeding

Kana. Kono, Chihiro. Tanikawa, Takeshi. Yanagita, Hiroshi. Kamioka and Takashi. Yamashiro*

*** Correspondence:** Takashi. Yamashiro: yamashiro@dent.osaka-u.ac.jp

1 Supplementary Figures

1.1 Supplementary Figure

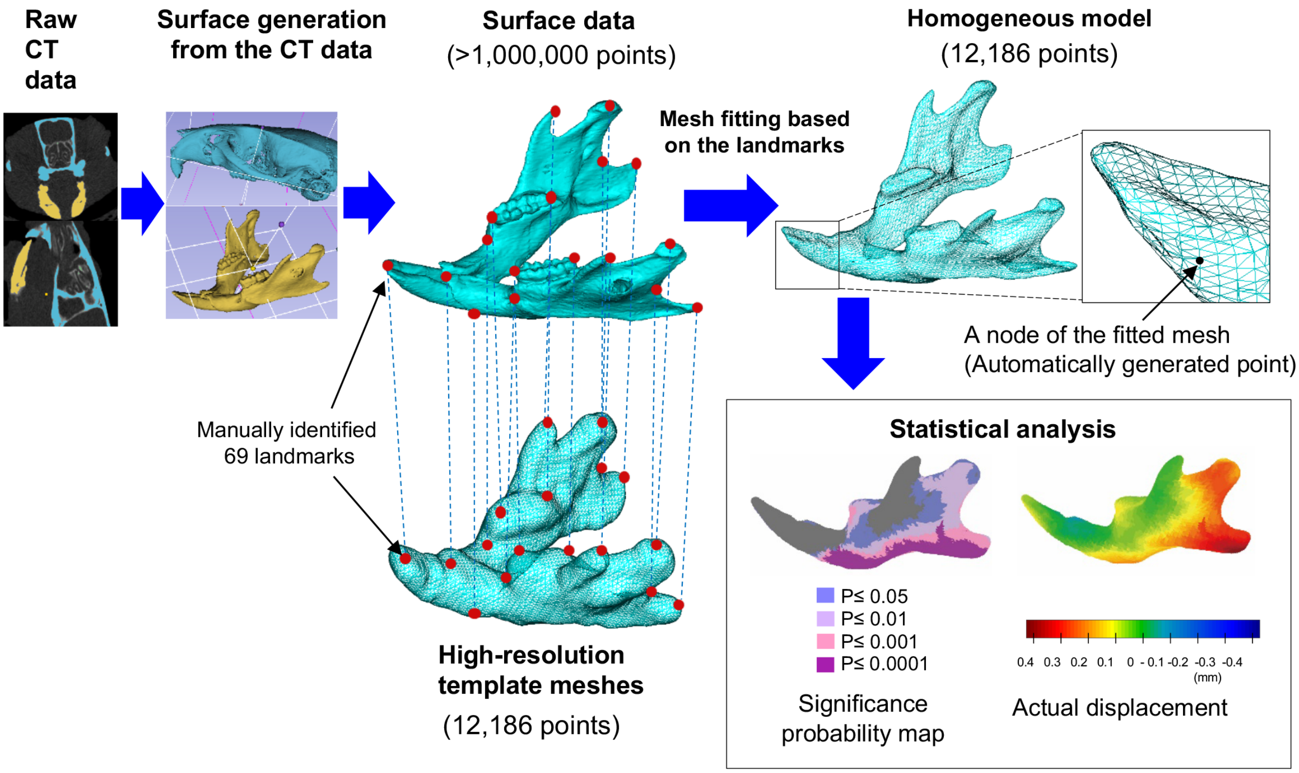

Supplementary Figure 1. Schematic illustration of the analyses employed in the present study. Firstly, surface generation from micro-CT data was performed. For each mandibular model, fitting high-resolution template meshes was performed based on the landmarks assigned to each 3D facial image. This method automatically generated a homogeneous model consisting of 12,186 points (i.e. nodes of the fitted mesh) on the wire mesh for each model. Finally, the coordinate values of each corresponding point on the wire mesh were used to generate color maps representing the actual displacement and significance of differences.

1.2 Supplementary Figure

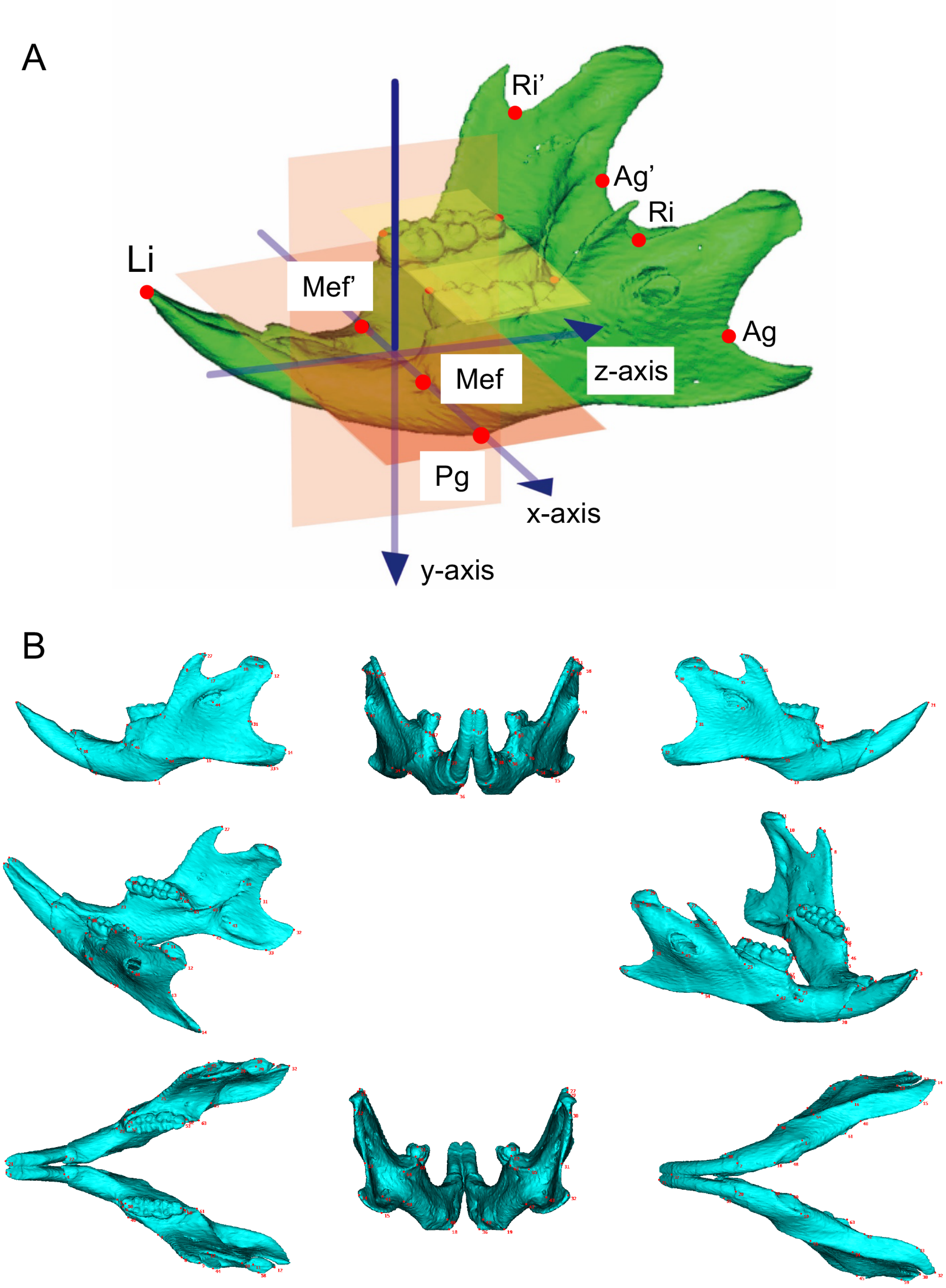

Supplementary Figure 2. The coordinate system (A) and 69 landmarks employed in the present study (B). The sagittal plane was defined by the midpoint at the center of gravity of Ri, Ii, Ag and Pg on the right side and of their counterparts, which were the points with the most accurate reproducibility (3.1-51.2 μm). The axial plane was defined as the molar occlusal plane (reproducibility of the anterior point of the molars = 33.9 μm; reproducibility of the posterior point of the molars = 50.0 μm). The midpoint of the mental foramen (Mef; reproducibility = 4.1 μm) was set as the origin. Ri indicates the most depressed point of the posterior coronoid process; Li, the most prominent point between the incisal edges of the lower incisors; Ag, an intersecting point between the mandibular bone and the distal surface of the lower third molar; and Pg, the most inferior contour of the lower border of the mandible, adjacent to the incisors.

1.3 Supplementary Figure

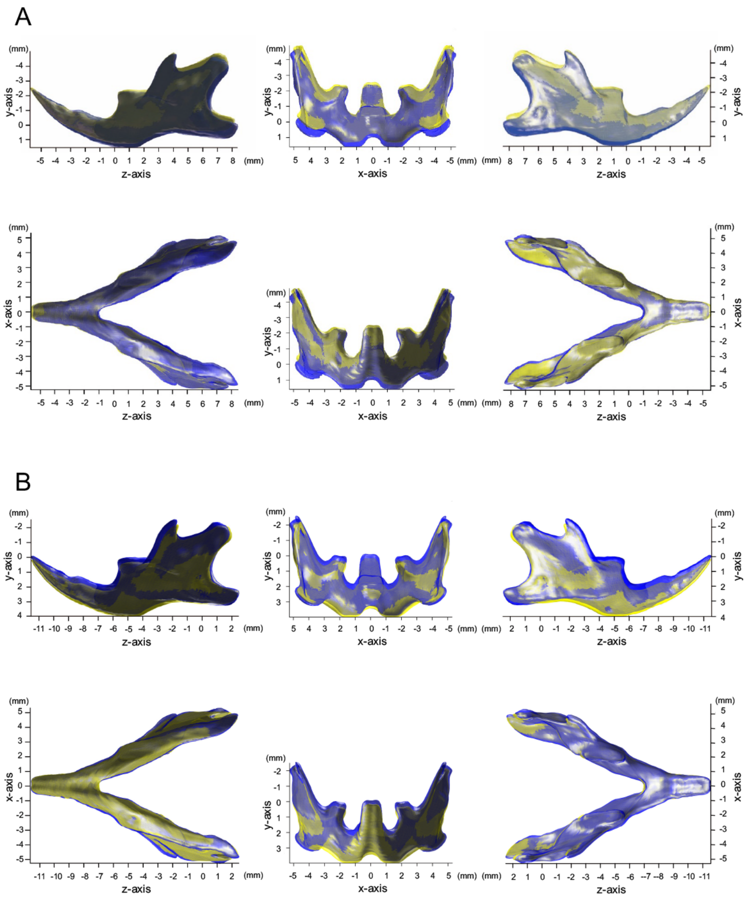

Supplementary Figure 3. Superimposed averaged images at the mental foramen (A) and at the mandibular foramen (B) parallel to the occlusal plane.

1.4 Supplementary Figure

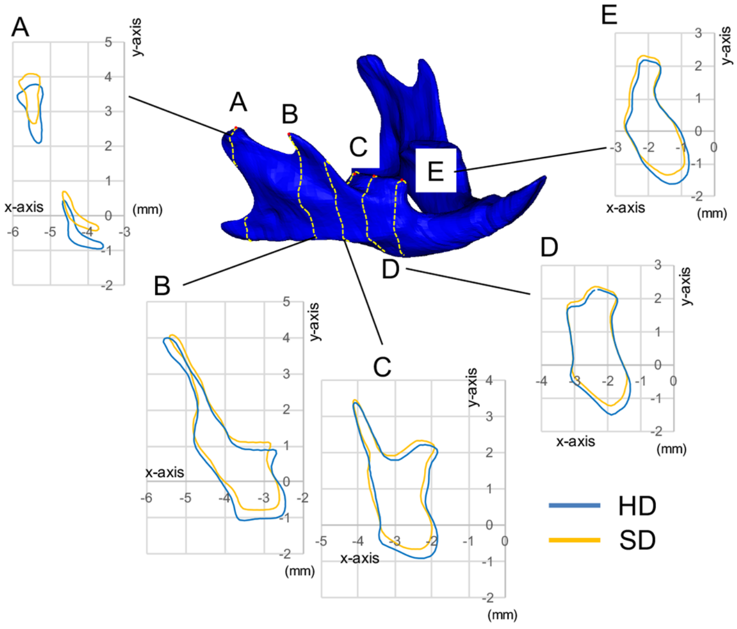

Supplementary Figure 4. Averaged coronal sectional line at the condylar process (A), at the coronoid process (B), posterior part of the molar (C), middle part of the molar (D) and anterior part of the molar (E).

1.5 Supplementary Figure

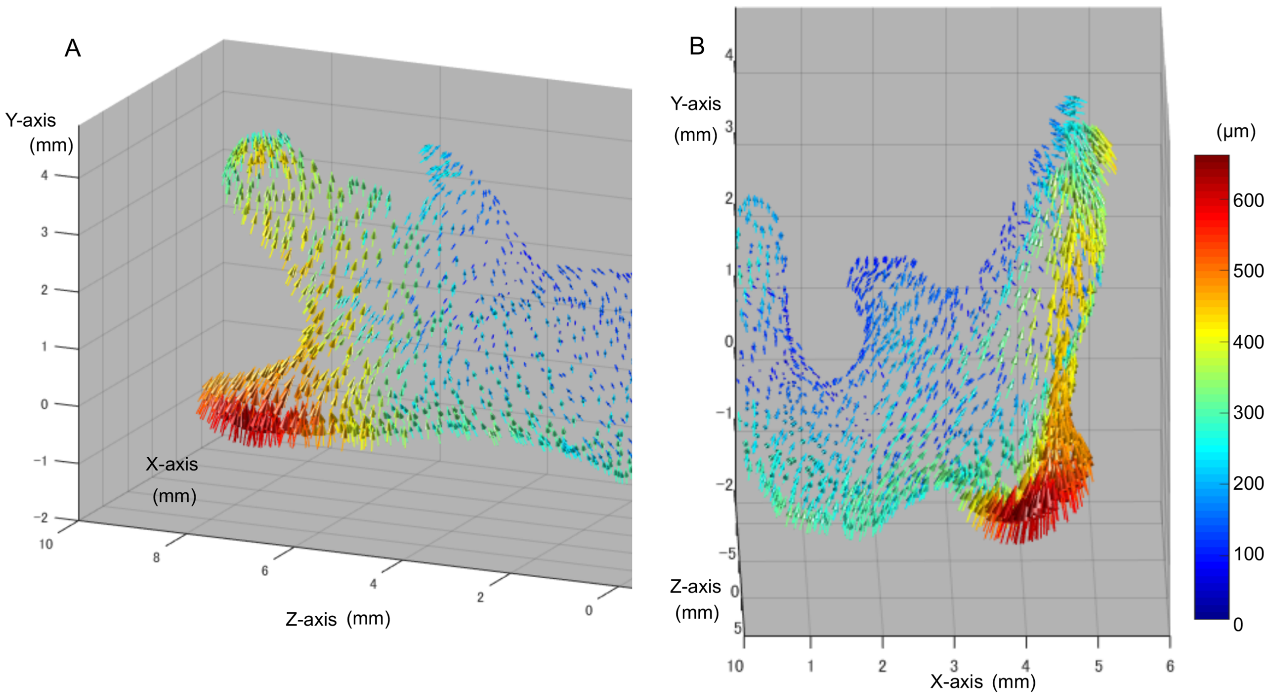

Supplementary Figure 5. Vectors from the average mesh points of the HD (arrow base) to those of the SD group (arrow apex). Greater scalar values are indicated by red and smaller values by blue. (A) Lateral view; (B) Back view.
